# Targeting caspase-6 and caspase-8 to promote neuronal survival following ischemic stroke

**DOI:** 10.1038/cddis.2015.272

**Published:** 2015-11-05

**Authors:** A P Shabanzadeh, P M D'Onofrio, P P Monnier, P D Koeberle

**Affiliations:** 1 Division of Anatomy, Department of Surgery, University of Toronto, Toronto, Canada; 2 Toronto Western Research Institute, Toronto Western Hospital, Toronto, Canada; 3 Graduate Department of Rehabilitation Science, University of Toronto, Toronto, Canada; 4 Departments of Physiology, University of Toronto, Toronto, Canada

## Abstract

Previous studies show that caspase-6 and caspase-8 are involved in neuronal apoptosis and regenerative failure after trauma of the adult central nervous system (CNS). In this study, we evaluated whether caspase-6 or -8 inhibitors can reduce cerebral or retinal injury after ischemia. Cerebral infarct volume, relative to appropriate controls, was significantly reduced in groups treated with caspase-6 or -8 inhibitors. Concomitantly, these treatments also reduced neurological deficits, reduced edema, increased cell proliferation, and increased neurofilament levels in the injured cerebrum. Caspase-6 and -8 inhibitors, or siRNAs, also increased retinal ganglion cell survival at 14 days after ischemic injury. Caspase-6 or -8 inhibition also decreased caspase-3, -6, and caspase-8 cleavage when assayed by western blot and reduced caspase-3 and -6 activities in colorimetric assays. We have shown that caspase-6 or caspase-8 inhibition decreases the neuropathological consequences of cerebral or retinal infarction, thereby emphasizing their importance in ischemic neuronal degeneration. As such, caspase-6 and -8 are potential targets for future therapies aimed at attenuating the devastating functional losses that result from retinal or cerebral stroke.

Stroke is the second-leading cause of disability and death in high-income countries.^1^ Thromboembolism, the physical blockage of a cerebral blood vessel, is a major cause of stroke.^2^ The bulk of ischemic episodes occur by occlusion of the middle cerebral artery (MCA) and its branches.^3^ Cerebral ischemia causes neuronal energy depletion and programmed cell death (apoptosis), both of which are facilitated by intermediate factors such as the release of excess excitatory amino acids,^4^ reactive oxygen species,^5^ free-radical formation, and inflammation.^6^

The majority of cerebral infarcts in humans originate from previously formed thrombi that detach from damaged carotid arteries and become lodged in branches of the MCA. Cerebral ischemia can be experimentally induced by injecting either a heterogeneous or an autologous pre-formed clot into the MCA. Thromboembolic stroke models are valuable in studying ischemic infarction because they recapitulate the hallmark symptoms of human cerebrovascular disease.^7, 8^ Moreover, thromboembolic-induced stroke shows predictable changes in blood flow and a more consistent degree of infarct distribution, relative to other models of middle cerebral artery occlusion (MCAO).^8, 9^

Retinal ischemia is also a common cause of visual impairment and blindness.^10^ Retinal ischemia induced by ligation or clamping of the ophthalmic artery is a reproducible model of CNS stroke that is highly amenable to experimental manipulations.^10, 11^ As the retina is an extension of the diencephalon, retinal blood vessels share similar anatomical and physiological properties with those in the brain, and possess a blood–retinal barrier analogous to the blood–brain barrier.^12^ Following the induction of retinal ischemia, ~50% of retinal ganglion cells (RGCs) die within the first 2 weeks after stroke.^13^

Cysteine-aspartic proteases (caspases) are a family of enzymes that orchestrate apoptosis, necrosis, and inflammation.^14, 15^ They are first synthesized as pro-caspases (zymogens) that consist of a prodomain, a small subunit (~p10 kDa) and a large subunit (~p20 KDa). Caspase-6 (CASP6) activation requires proteolytic processing (cleavage) of the zymogen into ~p10 and ~p20 fragments.^14, 16^ Caspase-8 (CASP8) activation occurs by dimerization, which causes a conformational change of the zymogen.^17^ Caspases orchestrate cell death in many neurodegenerative conditions: CASP6-dependent axon degeneration has been shown to contribute to Alzheimer's disease pathology,^15, 18^ and neurodegeneration associated with Huntington's disease,^19^ in several experimental models.^15, 18^ Furthermore, CASP8 promotes apoptosis induced by a Parkinson-associated mutation in leucine-rich repeat kinase 2.^20, 21^

Owing to early findings that caspases -3 and -9 were not involved in axonal degeneration,^22^ CNS axon degeneration was believed to be caspase-independent; however, it has been discovered that CASP6 is required for neuronal axon degeneration *in vitro*.^18^ Furthermore, we have shown a prominent role for CASP6 and CASP8 in RGC apoptosis and regenerative failure after optic nerve transection or optic nerve crush.^20^ In these injury models, CASP6 appears to activate CASP8 in injured RGCs and the inhibitory peptides Z-VEID-FMK and Z-IETD-FMK confer significant neuroprotection, while promoting axon regeneration in the crushed optic nerve.^20^ More recently, it was shown that CASP8 mRNA levels were increased in the ischemic cortex following MCAO.^23^ Consequently, we chose to examine the neuroprotective effects of CASP6 or CASP8 inhibition following cerebral or retinal ischemic injury, under normothermic conditions.

## Results

### Caspase-6 and caspase-8 inhibition promote retinal ganglion cell survival after ischemia

**Figure 1:**
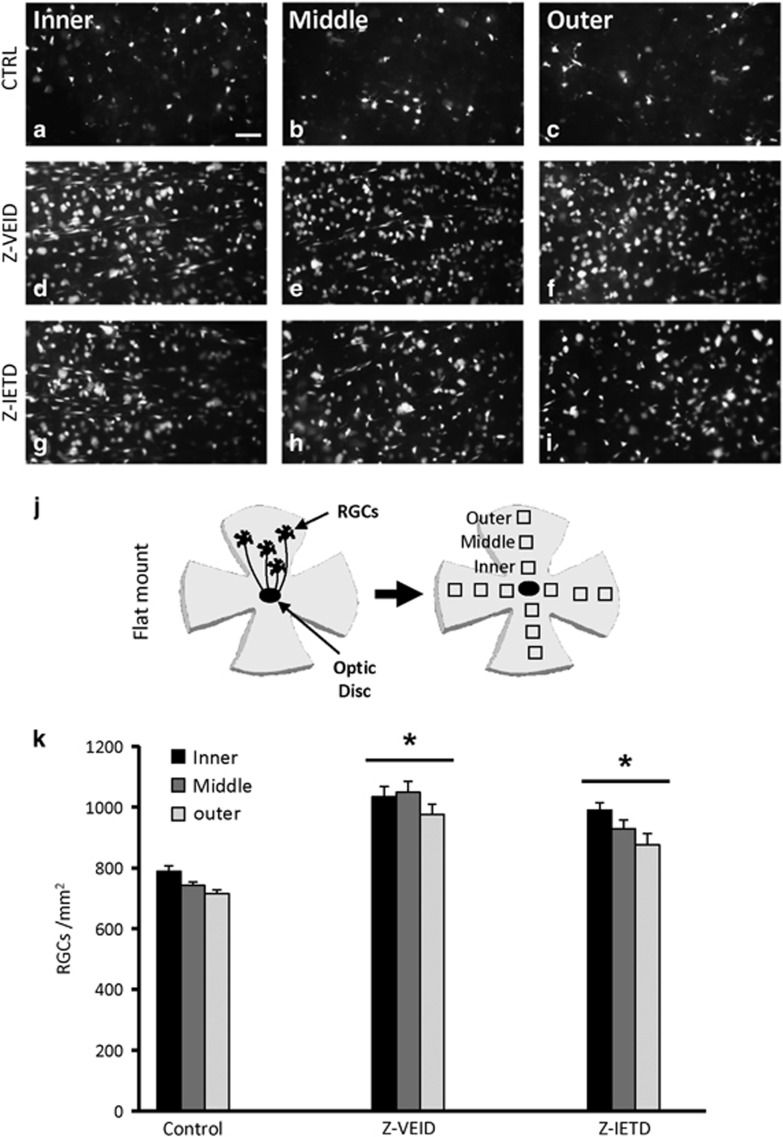
Caspase inhibition promotes RGC survival after ophthalmic artery ligation. (**a**–**i**) Epifluorescence micrographs of flat-mounted retinas showing Fluorogold-labeled RGCs at 14 days following ophthalmic artery ligation and various treatments (**a**–**c**) control retinas (*n*=6) had few surviving RGCs; (**d**–**f**) caspase-6 inhibition (Z-VEID-FMK; *n*=6) and caspase-8 inhibition (Z-IETD-FMK; *n*=6; **g**–**i**) increased RGC survival after retinal ischemia; (**j**) schematic of retinal flat-mounts, showing the three eccentric areas of RGC quantification (inner, middle, outer); (**k**) quantification of the density (cells/mm^2^) of surviving RGCs (±S.E.M.) at 14 days following ophthalmic artery ligation and treatment with caspase inhibitors. Z-VEID (caspase-6 inhibitor) or Z-IETD (caspase-8 inhibitor) significantly increased RGC survival (*P<0.001) after retinal ischemia. Scale bar, 50 *μ*m

We initially evaluated whether CASP6 or CASP8 inhibition could improve the survival of RGCs after a transient 30-min ophthalmic artery ligation. Ligation of the ophthalmic vessels produces a uniform ischemic injury in the inner retina, the location of RGC cell bodies (Figures 1a–k).^6^ We delivered Z-VEID-FMK (CASP6 inhibitor) or Z-IETD-FMK (CASP8 inhibitor) by intraocular injections at 3 and 10 days after ischemia, and the survival of Fluorogold pre-labeled RGCs was quantified at 14 days post ischemia. In the control group, the average density of RGCs was 748±15 cells/mm^2^ (Figures 1a–c). Administration of Z-VEID-FMK significantly increased RGC densities by 34% to 1019±35 cells/mm^2^ (*P*<0.001; Figures 1d–f). Similarly, Z-IETD-FMK increased RGC survival by 24% (931±30 cells/mm^2^; *P*<0.001; Figures 1g–i). Together, these results reveal that CASP6 and CASP8 have an important role in the degeneration of retinal ganglion cells after ischemia.

### siRNAs targeting caspase-6 or caspase-8 promote post-ischemic retinal ganglion cell survival

**Figure 2:**
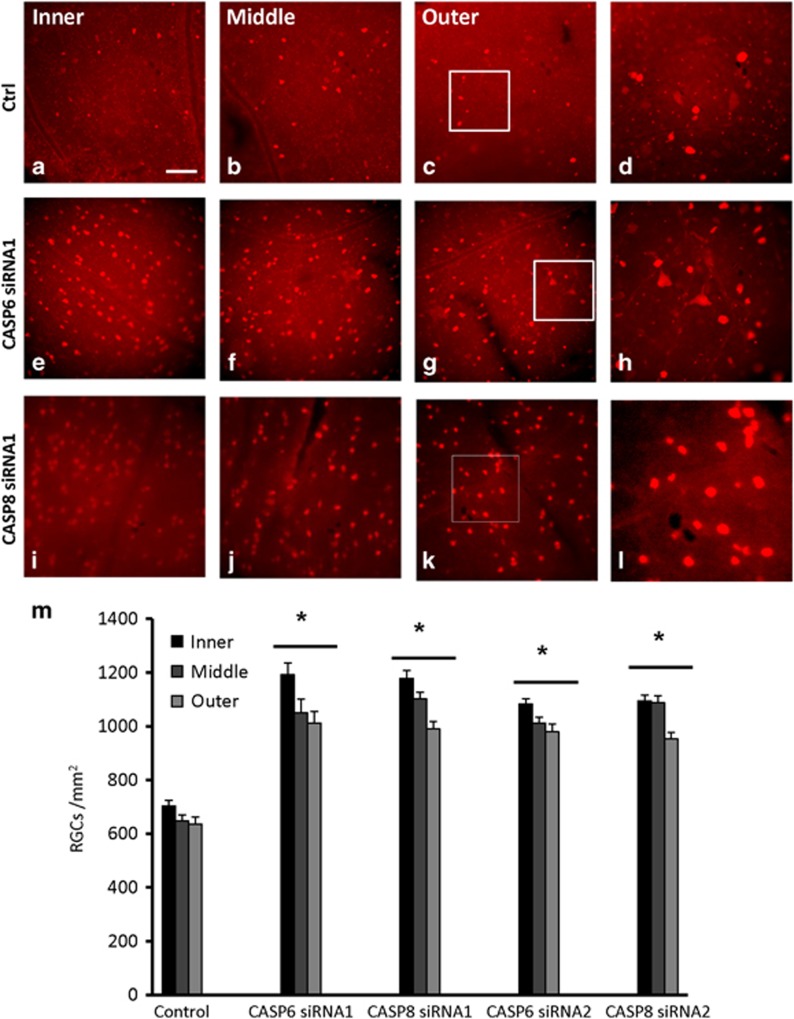
Caspase-6 and caspase-8 siRNAs promote RGC survival after retinal ischemia. (**a**–**l**) Epifluorescence micrographs of flat-mounted retinas showing RNA-binding protein with multiple splicing (RBPMS)-labeled RGCs at 14 days following ophthalmic artery ligation and various treatments. Images were taken in the inner, mid-periphery (middle), or periphery (outer) of the retina. Images in the right-hand column show magnified portions of the boxed regions in the third column (**a**–**d**) control retinas (*n*=6); (**e**–**h**) caspase-6 siRNA1 (CASP6 siRNA1; *n*=6) or caspase-8 siRNA1 (CASP8 siRNA1; *n*=6; **i**–**l**) increased RGC survival after retinal ischemia; (**m**) quantification of the density (cells/mm^2^) of surviving RGCs (±S.E.M.) at 14 days following ophthalmic artery ligation and treatment with caspase siRNAs. CASP6 siRNA1 and siRNA2 or CASP8 siRNA1 and siRNA2 significantly increased RGC survival (*P<0.001) after retinal ischemia; scale bar, 50 *μ*m

To evaluate the possible neuroprotective effects of CASP6 or CASP8 knockdown, we injected siRNAs into the vitreous chamber of the eye after ischemia. At 14 days post ischemia, control retinas, treated with a firefly luciferase siRNA, had a mean RGC density of 662±23 cells/mm^2^ (Figures 2a–d). In contrast, retinas treated with CASP6 siRNA1 (1084±45 cells/mm^2^) or CASP6 siRNA2 (1024±24 cells/mm^2^) showed a significant increase in RGC survival, compared with the control (*P*<0.001; Figures 2e–h). Similarly, CASP8 siRNA1 or CASP8 siRNA2 administration increased RGC survival by an average of 60% (1090±26 cells/mm^2^ and 1044±23 cells/mm^2^, respectively; *P*<0.001; Figures 2i–l). These results show that CASP6 or CASP8 siRNAs significantly protect RGCs from cell death induced by retinal ischemia (Figure 2m).

### Inhibition of caspase-6 and caspase-8 reduces brain infarction after MCAO

**Figure 3:**
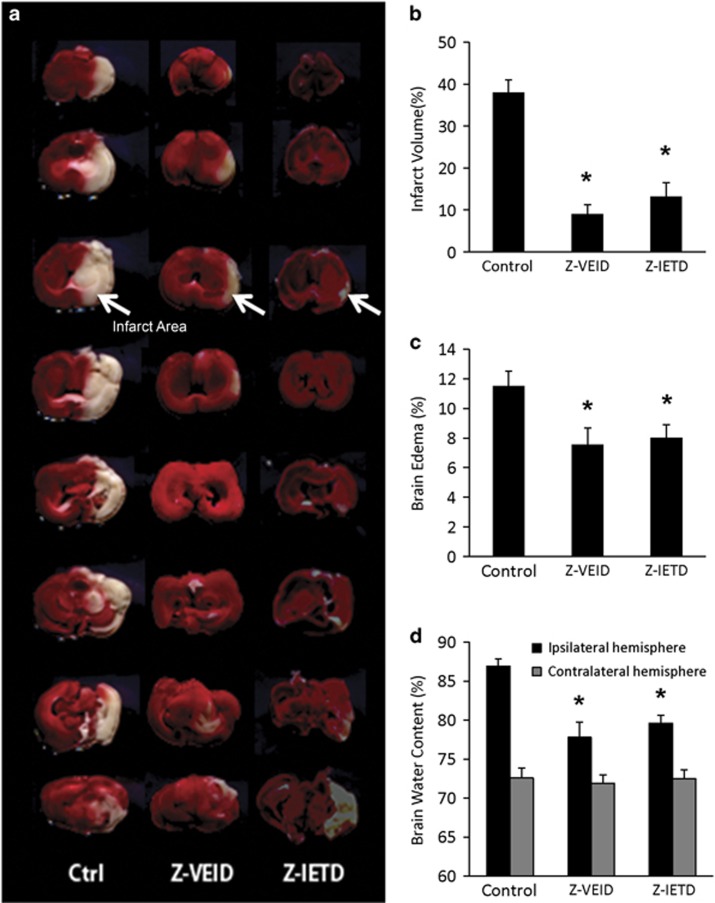
Brain edema and infarct volume at 48 h post ischemia are reduced by caspase inhibition. (**a**) Coronal brain slices showing the representative infarct area in a sample from control, caspase-6 inhibitor (Z-VEID)-, or caspase-8 inhibitor (Z-IETD)-treated brains. Brains were processed by TTC staining to detect the infarcted brain region (white area) within the remaining metabolically active brain tissue (red); (**b**) effect of control, caspase-6 inhibitor, or caspase-8 inhibitor treatment on infarct volume (percentage of unlesioned hemisphere); (**c**) effect of caspase inhibition on brain edema percentage relative to the uninjured (contralateral) hemisphere; (**d**) effect of caspase inhibition on mean brain water content of ipsilateral (injured) or contralateral (intact) cerebral hemispheres. Data in **b**, **c**, and **d** are presented as mean±S.E.M. *P<0.05 between control and experimental group; *n*=7 each group

**Figure 4:**
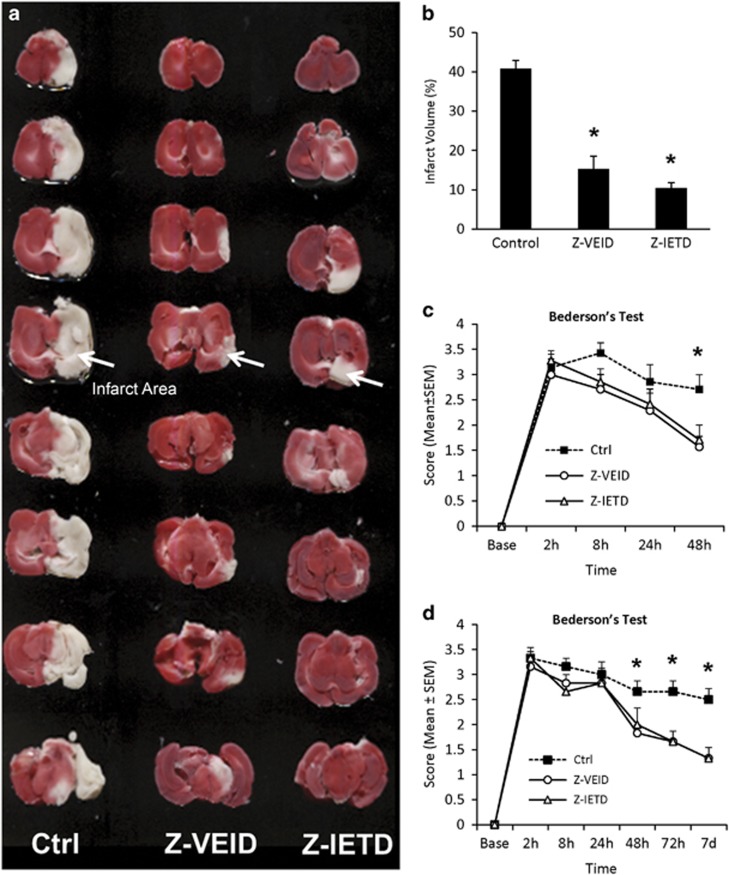
CASP6 or CASP8 inhibition reduce infarct volume and neurological deficits at 2 and 7 days post ischemia. (**a**) Coronal brain slices showing the representative infarct area in a sample from control, caspase-6 inhibitor (Z-VEID)-, or caspase-8 inhibitor (Z-IETD)-treated brains at 7 days after stroke. Brains were processed by TTC staining to detect the infarcted brain region (white area) within the remaining metabolically active brain tissue (red); (**b**) effect of control, caspase-6 inhibitor, or caspase-8 inhibitor treatment on infarct volume at 7 days after MCAO (percentage of unlesioned hemisphere); (**c** and **d**) Neurological deficits after stroke were assessed using the Bederson Scoring System. Graph depicts the Bederson scores for each of the three groups: control (Ctrl), caspase-6 inhibitor (Z-VEID), caspase-8 inhibitor (Z- IETD); (**c**) Bederson Scores were evaluated at baseline, 2, 8, 24, and 48 h after MCAO in the 48 h cohort; (**d**) Bederson Scores were evaluated at baseline, 2 h, 8 h, 24 h, 48 h, 72 h, and 7 days after MCAO, in the 7-day cohort. The neurological scores improved in both the treatment groups at 2 days after embolization. Data are presented as mean±S.E.M., *P<0.05 between control and experimental group; *n*=7 each group

As CASP6 or CASP8 inhibition were neuroprotective following retinal ischemia, we tested the effect of these interventions on brain infarction in a thromboembolic model of MCAO (Figures 3 and 4). Intravenous delivery of Z-VEID-FMK or Z-IETD-FMK significantly reduced brain infarct volume relative to control at 48 h and 7 days after MCA occlusion (Figures 3a and 4a). Compared with the control group, infarct volume was reduced by 62 and 52% (at 48 h post stroke) or 64 and 70% (at 7 days post stroke) in the ischemic rats that received Z-VEID- FMK or Z-IETD-FMK, respectively (*P*<0.001; Figures 3b and 4b).

### Caspase-6 or caspase-8 inhibition reduces brain edema and neurological deficits

The effect of CASP6 or CASP8 inhibition on brain edema was assessed by comparing the relative volumes of brain tissue between the infarcted and non-infarcted hemispheres of the brain. Brain edema was significantly reduced following intravenous delivery of either Z-VEID-FMK or Z-IETD-FMK: brain edema in control, Z-VEID-FMK, or Z-IETD-FMK groups was 11.5±1.02%, 7.56±1.12%, or 8.03±0.87%, respectively, at 48 h after MCA occlusion (Figure 3c).

We also measured the absolute and relative water content of the ipsilateral (ischemic) and contralateral (non-ischemic) hemispheres to further evaluate cerebral edema. The water content of the ipsilateral hemisphere in the control group was 86.92±0.92%. CASP6 or CASP8 inhibition reduced the relative water content of the ipsilateral hemisphere as compared with the control group: following treatment with Z-VEID-FMK or Z-IETD-FMK, the relative brain water content in the ipsilateral hemisphere was 77.79±1.93% or 79.59±1.04%, respectively (Figure 3d). The water content of the contralateral hemisphere did not change significantly among the groups. These findings provide further evidence that CASP6 or CASP8 inhibition reduces the edematogenic effect induced by MCAO.

Neurological scores were used to assess functional deficits after cerebral ischemia. Neurological scores were recorded before MCA occlusion and at 2, 8, 24, and 48 h afterwards (Table 1). At 2 and 8 h after MCA occlusion, all animals showed significant motor deficits, with median scores of 3 for all the groups (control, Z-VEID-FMK, and Z-IETD- FMK). At 48 h after MCA occlusion, neurological scores were significantly improved by Z-VEID-FMK or Z-IETD-FMK (both *P*<0.05), whereas no difference was evident at 24 h (Figure 4c). Similar experiments were carried out to evaluate neurological scores at 3 and 7 days after ischemia. At 3 days and 7 days after MCA occlusion, neurological scores were significantly improved by Z-VEID-FMK or Z-IETD-FMK (Figure 4d, both *P*<0.05). This shows that CASP6 or CASP8 inhibition reduces the neurological deficits that are associated with MCAO.

### Caspase-6 or caspase-8 inhibition reduces seizure activity (Racine's score)

Seizures were observed in three rats in the control group with average Racine's score of 4 at 2 h, and a score of 3 at 8, 24, or 48 h after MCAO in the 48-h cohort. Moreover, seizures were observed in four rats in the control group with average score of 4 at 2 h, and a score of 3 at 8, 24, 48, and 72 h (in the 7-day cohort). In contrast, no seizure activity was present in the CASP6 inhibitor-treated group, whereas one animal in the CASP8 inhibitor-treated group showed post-ischemic seizures at 2, 8, and 24 h in the 48-h cohort. One additional CASP8 inhibitor-treated animal showed seizure activity at 2, 8, 24, and 72 h in the 7-day study. The overall mortality rate within these groups was 7.1% (3/42) between 2 and 9 h after stroke.

### Effects of caspase-6 or caspase-8 inhibitors on endogenous caspase activation

**Figure 5:**
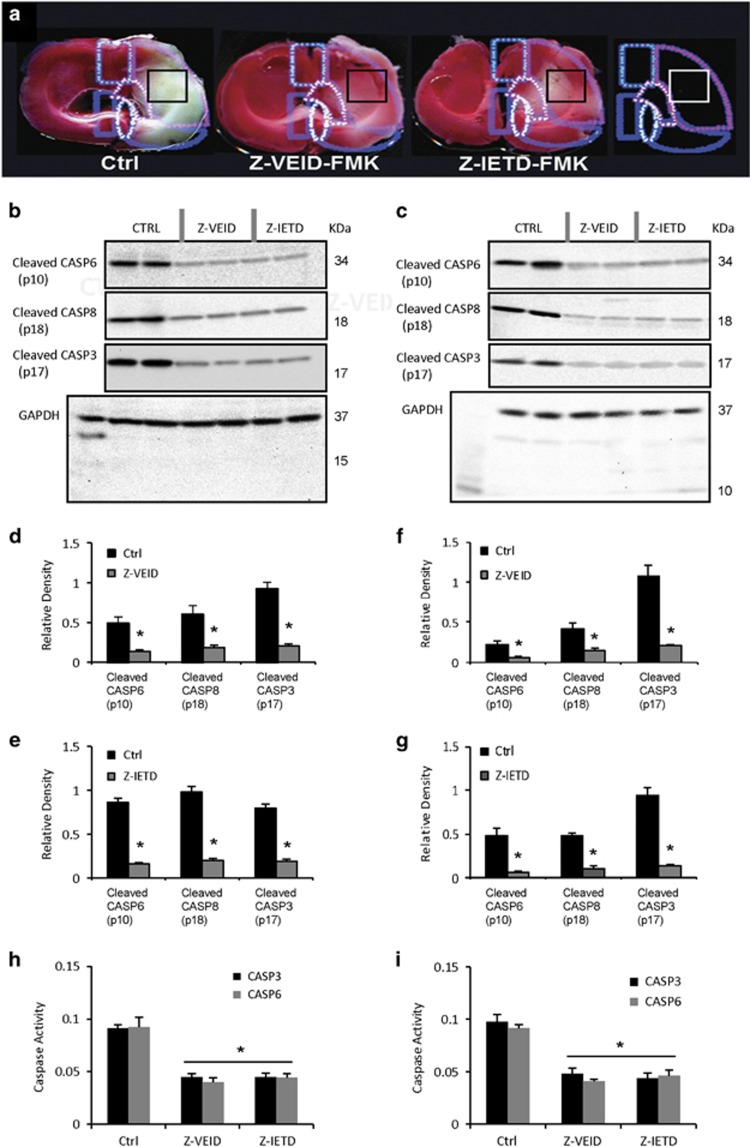
Effect of caspase-6 or -8 inhibition on caspase-3 activation after cerebral ischemia. (**a**) Two millimeter-thick coronal sections of rat brain stained with 2, 3, 5-triphenyletrazolium chloride (TTC) solution. Individual schematic representations of each group showing the location of tissue sampling (black square) for western blot analysis: control (DMSO; *n*=4), caspase-6 inhibitor (Z-VEID-FMK; *n*=4), and caspase-8 inhibitor (Z-IETD-FMK; *n*=4); (**b** and **c**) western blot analysis of brain samples (**b**) and whole retina (**c**) following control, caspase-6 inhibitor, or caspase-8 inhibitor delivery. The bands corresponding to the cleaved caspase-6 p10 subunit, cleaved caspase-8 p18 subunit and cleaved caspase-3 p17 are shown, with the corresponding GAPDH loading control at the bottom. For each treatment group, two lanes were loaded with lysate from the same tissue sample (Z-VEID, Z-IETD, or control DMSO); (**d**–**g**) quantification of cleaved caspase-6 (p10) subunit, cleaved caspase-8 (p18) subunit and cleaved caspase-3 (p17) levels at 48 h after embolization. Band intensity was normalized to the amount of GAPDH in each sample. Results are expressed as the mean of three separate brains±S.E.M. Z-VEID and Z-IETD treatment significantly reduced the amount of activated caspase-6 (p10), caspase-8 (p18), and caspase-3 (p17) at 48 h following thromboembolic ischemic injury; (**h** and **i**) Caspase activity assay showing CASP3 and CASP6 activity, based on cleavage of a colorimetric substrate in brain samples (**h**) and whole retinas (**i**). CASP3 and CASP6 activity was significantly decreased in rats treated with Z-VEID-FMK or Z-IETD-FMK compared with control (*n*=4 per group, *P<0.001)

After showing that CASP6 or CASP8 inhibition ameliorated the neuropathological consequences of ischemia, we evaluated whether these interventions affect caspase activation in the brain and retina. To do so, CASP6, CASP8, and caspase-3 (CASP3) western blots were performed on brain or whole retinal lysates to detect the cleaved p10 subunit of active CASP6, the cleaved p18 subunit of active CASP8, or the cleaved large subunit (p17) of CASP3 (Figures 5a–g). Brain samples were taken from the infarcted right cerebral hemisphere, as indicated in Figure 5a. As expected, CASP6 inhibition reduced the level of cleaved CASP6 p10 in whole retina and brain lysates (Figures 5b and c). Furthermore, reduced cleavage of CASP3 and CASP8 was observed after CASP6 inhibition (*P*<0.001; Figures 5b, d, and e), suggesting that CASP6 has a role in CASP3 and CASP8 activation. Similar experiments were carried out to evaluate whether CASP3 or CASP6 activation was dependent on CASP8: at 2 days after cerebral ischemia or 14 days after retinal ischemia, there was a significant reduction in the levels of CASP3 and CASP6 cleavage products in Z-IETD-FMK-treated rats (*P*<0.001; Figures 5c, f, and g). This shows that CASP8 is involved in the activation of CASP3 and CASP6 during cerebral and retinal ischemic injury. As expected, CASP8 inhibition also reduced the level of cleaved CASP8 p18 in both retinal and cerebral samples (Figures 5b and c). We then measured the activity of CASP3 and CASP6 in brain samples (Figure 5h) and whole retina lysates (Figure 5i), following CASP6 or CASP8 inhibition. A colorimetric caspase assay showed that CASP3 and CASP6 activity were significantly decreased in rats treated with Z-VEID-FMK or Z-IETD-FMK, compared with controls (*P*<0.001).

### Intravenous administration of caspase-6 and -8 inhibitors increases NF-200 levels after MCAO

**Figure 6:**
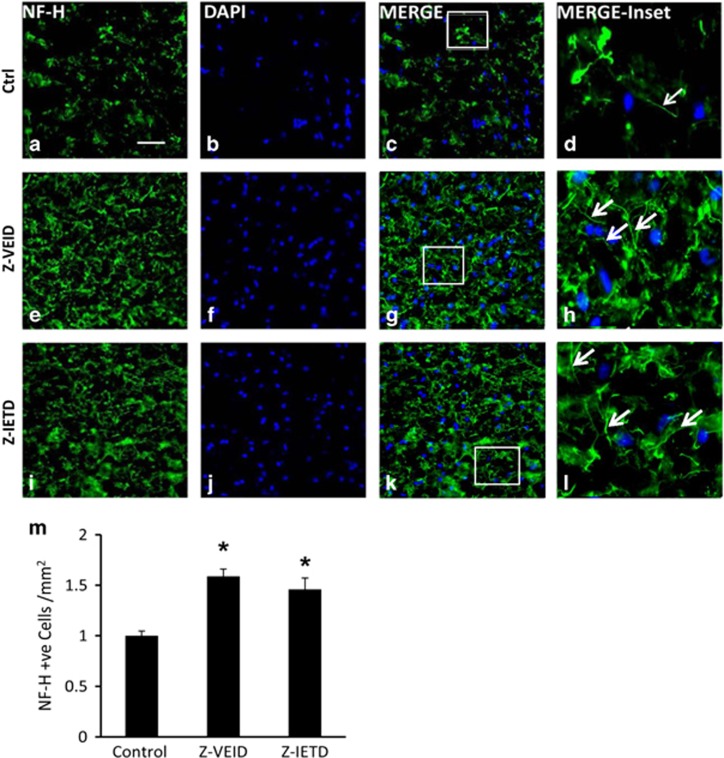
Effect of caspase-6 or caspase-8 inhibition on brain neurofilament levels. Intravenous administration of a caspase-6 or caspase-8 inhibitor increased the relative number of NF-200 immunopositive cells after MCAO. (**a**–**l**) Fluorescence micrographs of peri-infarct area of the injured hemisphere. Columns (left to right) show NF-200 (NF-H) immunoreactivity, DAPI labeling, a merge of the two preceding images, and a higher magnification view of the inset white box in the merged images. Control samples showed sparse neurofilament immunoreactivity (**a**–**d**), whereas samples treated with a caspase-6 inhibitor Z-VEID-FMK (**e**–**h**) or caspase-8 inhibitor Z-IETD-FMK (**i**–**l**) showed increased levels of NF-200 after MCAO. Arrows in **h** and **l** indicate the clear apposition and integrity of neurofilament (NF-H) immunoreactivity in the peri-infarct region of injured cerebral hemisphere compared with the arrow in **d** (control) after ischemia; (**m**) quantification of the relative mean number of NF-200-positive cells/mm^2^ (±S.E.M.) following MCAO. Z-VEID or Z-IETD significantly increased the number of NF-200-positive cells (*P<0.01) at 7 days after stroke. Scale bar, 50 *μ*m. *n*=6 each group

NF-200 has a critical role in maintaining neuronal shape,^24, 25^ and acting as a facilitator of axonal transport.^26^ To assess neuronal integrity after stroke and CASP6 or CASP8 inhibition, NF-200 immunostaining was examined in the peri-infarct region of the injured cerebral hemisphere (Figures 6a–l). The relative mean number of NF-200-positive neurons was significantly increased after either Z-VEID-FMK (1.59±0.73) or Z-IETD-FMK (1.46±0.12) treatment compared with the control group (1.0±0.04; *P*<0.01; Figure 6m), at 7 days after ischemia.

### Caspase-6 and -8 inhibitors increase the number of proliferating cells after MCAO

**Figure 7:**
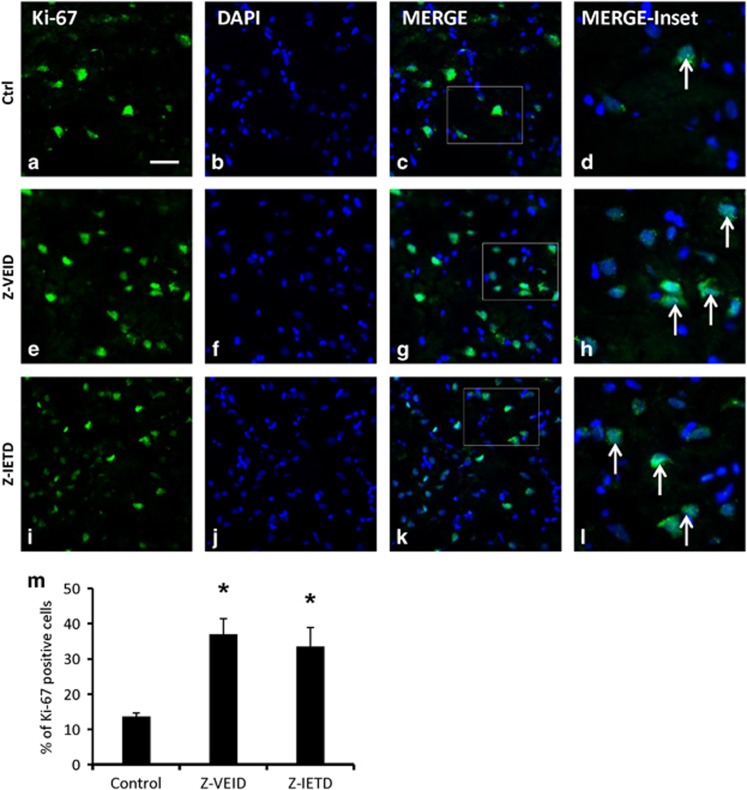
Effect of caspase-6 or caspase-8 inhibition on the number of proliferating cells after MCAO. Intravenous administration of a caspase-6 or caspase-8 inhibitor increased the percentage of Ki-67 positive cells after MCAO. (**a**–**l**) Fluorescence micrographs of peri-infarct area of the injured hemisphere. Columns (left to right) show Ki-67 immunoreactivity (proliferating cells), DAPI labeling, a merge of the two preceding images, and a higher magnification view of the inset white box in the merged images. Control samples showed scarce Ki-67 positive cells (**a**–**d**), whereas samples treated with a caspase-6 inhibitor Z-VEID-FMK (**e**–**h**) or caspase-8 inhibitor Z-IETD-FMK (**i**–**l**) had an increased percentage of Ki-67 positive cells after MCAO. Arrow in **d**, **h**, and **l** show Ki-67 positive proliferating cells in the peri-infarct region of the infarcted hemisphere; (**m**) quantification of the mean percentage of Ki-67 positive cells (±S.E.M.) following MCAO. Z-VEID-FMK or Z-IETD-FMK significantly increased the percentage of Ki-67 positive cells (*P<0.01) at 7 days after stroke. Scale bar, 50 *μ*m. *n*=6 each group

To assess cell proliferation after brain ischemia, we quantified the number of cells that were labeled with Ki-67 in the peri-infarct region of the ischemic hemisphere. Ki-67 is a nuclear marker that is expressed by proliferating cells, more noticeably after stroke.^27^ As shown in Figures 7a–l, the percentage of Ki-67 immunoreactive cells was significantly higher in the CASP6 or CASP8 inhibitor-treated groups (36.93±4.45 and 33.51±5.4, respectively) when compared with the control (13.5±0.99, *P*<0.01; Figure 7m).

## Discussion

To reduce apoptosis and neurological deficits after stroke, novel pharmacological approaches are needed to increase the capacity for regeneration and recovery in the CNS.^28^ In the present study, we examined the efficacy of peptide-based CASP6 or CASP8 inhibitors in abrogating the neurodegeneration associated with cerebral or retinal ischemia.

Our findings show that inhibition of CASP6 or CASP8 via intraocular delivery of Z- VEID-FMK or Z-IETD-FMK promotes RGC survival after transient retinal ischemia. Furthermore, RNA interference by intraocular delivery of CASP6 or CASP8 siRNAs enhanced RGC survival. The magnitude of the neuroprotective effect of siRNA delivery in the retinal ischemia model was comparable to that of Z-VEID-FMK or Z-IETD-FMK. These findings were then corroborated in a thromboembolic cerebral stroke model, where CASP6 and CASP8 inhibitors reduced infarct volume, increased the number of proliferating cells, and improved functional recovery after MCAO. Mechanistically, we showed that CASP6 or CASP8 inhibition (Z-VEID-FMK or Z-IETD-FMK) reduced the levels of cleaved procaspase-3 and procaspase-6 following cerebral ischemia. Taken together, these results demonstrate that CASP6 or CASP8 inhibitors have the potential to significantly attenuate the physiological consequences of both retinal and cerebral ischemic injury under normothermic conditions.

### Caspase-6 and -8 are key players in the neuropathology of retinal ischemia

Cleavage and activation of caspase-3, -8, and -9 are well-known hallmarks of RGC degeneration following optic nerve transection.^29, 30^ It has recently been discovered that CASP6 and CASP8 are required for neuronal axon degeneration *in vitro* and *in vivo.*^18, 20^ Moreover, caspase-6 and -8 levels negatively correlate with RGC survival following optic nerve crush and transection, and contribute to the regenerative failure of axons.^18^ We demonstrated that CASP6 is localized to RGC cell bodies in the ganglion cell layer.^20^ Furthermore, CASP6 and CASP8 appear to activate one another in a recurrent fashion in the injured retina. The present study illustrates that CASP6 inhibition reduces CASP3 cleavage in addition to the expected reduction in autolytic CASP6 activation in the injured cerebral cortex. Similarly, CASP8 inhibition reduced both CASP3 and CASP6 activation, suggesting that the cross-activation of these caspases is common in both the injured cerebrum and retina. It is likely that other caspases, aside from CASP8, also contribute to CASP6 activation in the retina because caspase-8 inhibition does not completely abolish CASP6 cleavage,^20^ or, as is demonstrated in the present study, CASP3 cleavage.

Several mechanisms may contribute to the beneficial effects of CASP6/8 inhibition in the ischemic retina. For example, it has been reported that Fas ligand binding to its death receptor induces apoptosis through the activation of CASP8, which in turn activates CASP3 and CASP9.^31^ CASP6 can also be activated by several death receptors.^18^ In accordance, CASP6 or CASP8 inhibition may antagonize pro-apoptotic signaling through a death receptor such as Fas/CD95, which has been shown to be involved in RGC apoptosis.^32^

CASP6 inhibitors have also been found to reduce the cleavage of microtubule-associated proteins such as TAU^18, 33^ and inhibit ROCK activation, independently of CASP3.^34^ ROCK is a well-known inhibitor of axon regeneration and promoter of cell death in RGCs.^35, 36^ Interestingly, the effects of CASP6 or CASP8 inhibition appear to be partially dependent on ROCK as additive effects on RGC regeneration were not observed with co-delivery of the ROCK inhibitor Y-27632.^20^

### Caspase-6 and -8 have an important role in cerebral ischemic pathology

Our data implicate CASP6 as an important mediator of ischemia-induced neuronal degeneration. It has been shown that CASP6 cleaves tubulin in the CNS after ischemic injury,^33, 37^ which may disrupt axon stability. The temporal activation of CASP6 in the stroke penumbra corresponds with the progression of axonal degeneration, which is a major contributor to cell death.^38, 39^ Activated CASP6 has been observed in neuronal processes and cell bodies after stroke,^40^ with distinct patterns of mRNA expression following the induction of cerebral ischemia. Moreover, genetic deletion of CASP6 provides improved neurological function and protects against neuronal process loss and neuronal death.^23^ In particular, cleaved CASP6 and CASP3 are both highly abundant in the penumbral region of the infarct,^40, 41^ where they may act in concert to promote programmed cell death. It is noteworthy that the levels of active CASP3 were reduced by CASP6 or CASP8 inhibition after MCAO in the present study, suggestive that CASP3 is activated downstream of both CASP6 and CASP8 in this model.

In the setting of focal ischemia, CASP8 expression has previously been observed in neurons after cerebral infarction,^42, 43^ and the present data point to an important role for CASP8 in the neuropathology of ischemia. CASP8 inhibition may protect CNS neurons via a number of different mechanisms: for example, CASP8 inhibition has been shown to minimize neurodegeneration in the inflamed brain by selectively killing activated microglia.^44^ Another possible mechanism is the modulation of TNF death-receptor signaling: TNF is involved in systemic inflammation, and it has been shown that, in certain circumstances, its downstream signaling can lead to direct activation of CASP8.^45, 46^ In this series of events, activated CASP8 could then cleave downstream caspases, resulting in CASP3 activation and apoptosis.^45, 46^ Indeed, the administration of Z-IETD-FMK appears to prevent apoptosis in human leukemia cells by inhibiting CASP8-mediated activation of CASP3.^47^ In the present study, we similarly observed this effect after stroke *in vivo*.

Interestingly, systemic administration of either Z-IETD-FMK or Z-VEID-FMK masked the edematogenic effects of MCA occlusion. It is unclear how this occurs; however, one possible mechanism involves caspase-1 as an upstream regulator of CASP6-mediated cell death.^48^ It has been shown that inhibition of caspase-1 prevents the release of active interleukin-1*β* and Interleukin 18, both of which are known to initiate inflammation and induce blood–brain barrier disruption and edema.^49, 50^ This would be expected to decrease the intracranial pressure, allowing greater blood flow to the areas surrounding the infarct, thereby improving neurological outcome. It is also possible that CASP6 or CASP8 inhibition has antithrombotic activity through the prevention of downstream CASP3 activation: for example, CASP3 inhibition has been shown to enhance clot lysis, thereby reducing platelet aggregation.^51^

Our data show that Z-IETD-FMK or Z-VEID-FMK reduce cerebral infarct volume and improve neurological scores. On the basis of Ki-67 staining, we showed an increase in the number of proliferating cells in the peri-infarct area, following CASP6 or CASP8 inhibition in the MCAO model. Furthermore, the expression of NF-200 in the peri-infarct region was augmented after caspase inhibition. NF-200 is the active (phosphorylated) form of the heaviest subclass of neurofilament subunits, also referred to as NF-H.^52, 53^ It is among the most phosphorylated proteins in the brain^54, 55^ and contains a sequence of amino acids that is important in the formation of the parallel structure of the neurofilament bundle.^56^ NF-200 in the cytoskeletal complex regulates inter-filament spacing and axonal caliber.^24, 57, 58, 59, 60^ As such, NF-200 is particularly abundant in neurons with large diameter axons, such as motor neurons, where fast impulse conduction velocities are indispensable.^58, 61, 62^ NF-200 may also mediate interactions with other cytoskeletal components, particularly microtubules that are involved in intracellular transport,^63, 64^ and essential for neuronal survival and function.^65^ Accordingly, the increased NF-200 immunoreactivity that we observed following CASP6 or CASP8 inhibition is suggestive of enhanced neuronal preservation after MCAO.

## Conclusions

We have shown that caspase-6 or -8 inhibition decreases the neuropathological consequences of cerebral and retinal infarction. As such, the development of therapeutics capable of targeting caspase-6 or -8 has potential for ameliorating the devastating loss of function resulting from retinal or cerebral stroke.

## Materials and Methods

### Retinal stroke model

This study used female Sprague-Dawley rats (Charles River, Senneville, QC, Canada), weighing 250–300 g, that were kept in a pathogen-free environment and cared for according to the Canadian Council on Animal Care. Retinal ischemia was carried out as previously described.^66, 67^ One week before ischemia, animals received stereotaxic injections of 2% Fluorogold into the superior colliculus, the brain target of RGCs, to retrogradely pre-label all RGCs in the retina for future quantification.

Seven days after RGC labeling, retinal ischemia was induced. Animals were placed in a stereotaxic frame and ventilated with isoflurane (2% 0.8 l/min O_2_) through a gas anesthesia mask. The optic nerve and ophthalmic vessels were accessed through the orbit of the eye via a superior route, after which the dural sheath surrounding the optic nerve was cut longitudinally, to avoid damaging the retinal vasculature. The optic nerve was lifted from the meningeal sheath, after which the surrounding dura and ophthalmic artery were ligated for 30 min. The orbital contents were then returned to their original locations and the initial incision was closed. Following surgery, the animals were kept at 37 °C underneath a heat lamp. They were given Ketoprofen (5 mg/ml, dosage for rats: 0.1 ml/100 g bodyweight) and sterile saline to ease postsurgical recovery.

### Intraocular injections

To test the effect of caspase-6 or -8 inhibitors on RGC survival in the ischemic retina, animals were randomized and divided into three groups (*n*=6 each): a control group that received intraocular injections of DMSO vehicle, a treatment group that received intraocular injections of a caspase-6 inhibitor (Z-VEID-FMK; 2 *μ*l of 25 mM stock dissolved in DMSO), and another treatment group that received intraocular injections of a caspase-8 inhibitor (Z-IETD-FMK; 2 *μ*l of 25 mM stock dissolved in DMSO). These inhibitory peptides are expected to have a half-life in the vitreous humor of ~19 h, also being able to cross the blood–brain barrier.^68, 69, 70^

Intraocular injections (4 *μ*l) of experimental or control vehicle solutions were administered at 3 days and 10 days after retinal ischemic injury; the onset of RGC apoptosis occurs at 4–5 days after retinal ischemia.^20^ RGC survival was quantified at 14 days after ischemia. Intraocular injections were performed as previously described.^20, 71, 72^ Animals were anesthetized with 3.5% isoflurane and the cornea was anesthetized using Alcaine eye drops (Alcon) before intraocular injections. A pulled glass micropipette attached to a 10 *μ*l Hamilton syringe via a hydraulic coupling through PEEK tubing was used to deliver 4 *μ*l of a solution into the vitreous chamber of the eye, posterior to the limbus. Following injection, the cornea was covered with ophthalmic ointment to prevent desiccation.

### Quantification of RGC survival after injury

RGC survival after injury was quantified via two different methods: Fluorogold retrograde labeling or immunohistochemistry directed against RNA-binding protein with multiple splicing (RBPMS) staining as previously detailed.^72, 73^

Epifluorescence imaging was used to visualize Fluorogold pre-labeled RGCs after retinal ischemia. Following retrograde labeling with Fluorogold, RGC cell bodies in the ganglion cell layer and axon fascicles in the nerve fiber layer of the retina were clearly visible upon imaging in a flat mount preparation. Animals were euthanized at 14 days after ophthalmic artery ligation, at which point the eyes were enucleated, dissected, and the retinas fixed in 4% paraformaldehyde for 1 h and then rinsed in PBS for 15 min. The retinas were then flat-mounted and coverslipped in 50 : 50 glycerol/PBS media for visualization. Fluorogold staining in RGCs was visualized with a fluorescence microscope using a wide band ultraviolet excitation filter and an Andor Neo sCMOS camera mounted on a Leica DM LFSA microscope. A Sutter Lambda XL illuminator (Quorum Technologies, Guelph, Canada) served as the light source with liquid light guide ensuring even field illumination. The density of RGCs was measured at three different distances from the central optic disk of the flat-mounted retinas: samples were taken from the inner (1/6 retinal eccentricity from the optic disk), mid-periphery (1/2 retinal eccentricity from the optic disk), and outer retina (5/6 retinal eccentricity from the optic disk) of the retinal quadrants. RGC densities (cells/mm^2^) were grouped by retinal eccentricity (inner, middle, outer) and expressed as mean±S.E.M.

RBPMS immunohistochemistry was performed on whole retinas. Retinas were incubated overnight at 4 °C in the primary antibody (Anti-RBPMS, 1 : 500, PhosphoSolutions, Aurora, CO, USA) that has been shown to label mammalian RGCs.^74^ The primary antibody was diluted in PBS containing 0.3% Triton X-100 and 3% normal serum. Following primary antibody incubation, whole retinas were rinsed three times for 15 min in PBS and incubated with FITC-labeled secondary antibody for 3 h at room temperature. Retinas were then rinsed three times for 15 min each time in PBS, flat mounted and coverslipped with 50 : 50 glycerol/PBS. Epifluorescence imaging was used to visualize and quantify RGCs as described in previous paragraph.

### CASP6 or CASP8 siRNA treatment after retinal ischemia

All siRNAs used in this study were synthesized at Integrated DNA Technologies Inc. (Coralville, IA, USA). The following CASP6 siRNA duplex sequences were used in the present study:

CASP6 siRNA1: 5′-rGrCrU rArGrG rArUrU rUrGrA rArGrU rGrArA rArUrG rCrUT T-3′

5′-rArArA rGrCrA rUrUrU rCrArC rUrUrC rArArA rUrCrC rUrArG rCrUrC-3′

CASP6 siRNA2: 5′-rGrCrA rArArG rArCrC rCrArG rGrUrG rCrArA rUrUrG rGrCA A-3′

5′-rUrUrG rCrCrA rArUrU rGrCrA rCrCrU rGrGrG rUrCrU rUrUrG rCrArG-3′

CASP8 siRNA duplex sequences used in the present study were:

CASP8 siRNA1: 5′-rGrCrA rArGrA rGrArG rUrGrA rGrUrC rArCrU rArArA rUrUC A-3′

5′-rUrGrA rArUrU rUrArG rUrGrA rCrUrC rArCrU rCrUrC rUrUrG rCrUrC-3′

CASP8 siRNA2: 5′-rGrArU rGrUrU rGrGrA rGrGrA rArGrA rCrArA rUrUrU rGrUC C-3′

5′-rGrGrA rCrArA rArUrU rGrUrC rUrUrC rCrUrC rCrArA rCrArU rCrCrC-3′

The control siRNA duplex sequences were directed against firefly luciferase:

5′-rCrUrA rGrArG rGrArU rArGrA rArUrG rGrCrG rCrCrG rGrGrC rCrUrU-3′

5′-rGrGrC rCrCrG rGrCrG rCrCrA rUrUrC rUrArU rCrCrU rCrUA G-3′

An intravitreal injection of 4 *μ*l of a 40 nmol solution of siRNA was administrated per rat eye after retinal ischemia. The effect of the control, CASP6, and CASP8 siRNAs was assessed by quantifying the number of RBPMS-labeled RGCs (Anti-RBPMS; PhosphoSolutions) in retinal whole mounts.

### Induction of thromboembolic focal cerebral ischemia

The effects of CASP6 or CASP8 inhibitors on ischemic brain injury in normothermic rats were studied after MCAO. Animals were randomly assigned to two series of cohorts. In the first series, we evaluated the effects of CASP6 or CASP8 inhibitors on brain infarction at 48 h after MCAO in three groups: control group (DMSO; seven rats), CASP6 inhibitor group (Z-VEID-FMK; seven rats), or CASP8 inhibitor group (Z-IETD-FMK; seven rats). In the second series, we evaluated the effects of CASP6 or CASP8 inhibitors at 7 days post stroke in three similar groups (*n*=7 per group). All inhibitors were dissolved in sterile DMSO. Z-VEID-FMK and Z-IETD-FMK were delivered at a dose of 1 mg/kg via intravenous (tail vein) injection. As the number of necrotic neurons increases most markedly during the 6 to 12 h interval after MCA occlusion^75^ and the time course of cortical apoptosis is less than 24 h after MCAO,^43^ the initial caspase inhibitor treatment was administered immediately after MCAO and repeated at 24 h in the present study.

For thromboembolic ischemia, a pre-formed blot clot was injected into the MCA via the internal carotid artery.^8, 76^ The rats were initially anesthetized with 3.0% isoflurane and then maintained with 1.5% isoflurane in O^2^, with a face mask during surgery. Body temperature was maintained at 37 °C with a heating pad for the duration of the surgery and via a heat lamp during the postoperative period until the animal was fully recovered from the anesthetic. A 1.5-cm longitudinal incision was made in the midline of the ventral cervical skin. The right common carotid artery (CCA), right internal carotid artery (ICA), and right external carotid artery (ECA) were exposed. The distal portion of the ECA was ligated and cut, and a modified polyethylene-10 catheter, filled with bovine thrombin (Thrombostat, Warner-Lambert Co., Morris Plains, NJ, USA), was introduced into the lumen of the right ECA via a small puncture. Ten microliters of blood were withdrawn into the catheter and retained for 15 min to allow formation of a clot. Once the clot was formed, the catheter was advanced 17 mm into the internal carotid artery until the tip was 1–2 mm away from the origin of the MCA. The pre-formed clot in the catheter was then injected, and the catheter was removed. The wound was then closed and the animals were moved to recovery cages.

### Quantification of brain infarct volume and edema

Quantification of infarct volume and edema was performed as previously described.^8, 76^ Briefly, the infarct volume was expressed as a percentage of the total volume of unlesioned left hemisphere. Brain edema was determined by calculating the volume difference between the two hemispheres and dividing by the volume of the unlesioned left hemisphere.

Two and 7 days after MCA occlusion, rats were euthanized by cervical dislocation. The brains were then removed and cooled in ice-cold saline for 5 min. A total of eight (2 mm thick) coronal sections were collected using a rat brain matrix, and stained using a 2% 2, 3, 5- triphenyltetrazolium chloride (TTC) solution at 37 °C. After TTC staining, the infarcted brain tissue appears white, whereas metabolically active areas of the brain are stained with a dark red precipitate. The stained brain sections were scanned with an Epson Perfection V300 flatbed scanner. The infarct volume and brain edema were calculated using the following formulae: infarct volume=(left hemisphere volume−(right hemisphere volume−measured infarct volume))/left hemisphere volume, edema=(right hemisphere volume−left hemisphere volume)/left hemisphere volume.

### Brain water content measurement

The anti-edematogenic effects of CASP6 and CASP8 inhibitors in the ischemic-injured hemisphere were studied in a separate experiment. Animals were randomly assigned to three groups as follows: control group (DMSO; *n*=7), CASP6 inhibitor group (Z-VEID-FMK; *n*=7), and CASP8 inhibitor group (Z-IETD-FMK; *n*=7). We used the wet–dry weight method to quantify cerebral edema.^77, 78^ The rats were killed after 48 h of reperfusion and the brain tissue was removed. Each hemisphere of brain tissue was weighed separately. The tissue was then dried at 110 °C for 24 h and the samples were weighed again. Both wet and dry weights were recorded, and the water content was expressed using the following equation: %Brain water content=100 × (wet weight−dry weight)/wet weight.

### Neurological deficits and seizure activity

Neurological deficits and seizure activity in each rat were evaluated at 2, 8, 24, and 48 h (in the 48 h cohort) and at 2, 8, 24, 48, or 72 h, and 7 days (in the 7-day cohort) following ischemic injury by an observer who had no knowledge of which procedure had been performed. Neurological deficits and seizure activities were classified with Bederson's and Racine's scoring systems.^79, 80^

Bederson's scoring system: 0, no observable deficit (normal); 1, forelimb flexion (moderate); 2, forelimb flexion plus decreased resistance to lateral push (moderate); 3, unidirectional circling (severe); 4, unidirectional circling plus decreased level of consciousness (severe).

Racine's scoring system: 0, no seizure activity (normal); 1, rhythmic mouth and facial movement (moderate); 2, rhythmic head nodding (moderate); 3, forelimb clonus (severe); 4, rearing and bilateral forelimb clonus (severe); 5, rearing and falling over (very severe).

### Caspase western blots

Activation (cleavage) of caspase-6 and caspase-8 was evaluated by western blot as previously described.^20, 67^ Animals were randomly assigned to three groups as follows: control group (DMSO; *n*=4), CASP6 inhibitor group (Z-VEID-FMK; *n*=4), and CASP8 inhibitor group (Z-IETD-FMK; *n*=4). The samples from each group were processed at 48 h (cerebral) and 14 days (retinal) after ischemia. A 0.5 cm^2^ brain sample from the lateral region of infarcted cerebrum or the whole ischemic retina was placed in 1 ml or 400 *μ*l of ice-cold SDS lysis buffer (2% SDS, 0.3% DTT, 10% glycerol in 40mMTris-Cl, pH6.8), respectively. The samples were then homogenized by ultrasonic disruption, and the remaining solutions were heated to 85 °C for 8 min, centrifuged (12 000 r.p.m., 12 min, 4 °C), and the protein samples were separated from the pelleted debris.

Total protein fractions were separated by SDS-PAGE on Bio-Rad (Mississauga, ON, Canada) TGX Gels (5–20%) and immunoblotted. After semidry electrotransfer to nitrocellulose membranes (0.2 *μ*m pore size), blots were blocked in 5% milk in Tris-buffered saline containing 0.1% Tween 20 (TBS-T) for 1 h at room temperature. Blots were then incubated in primary antibody solutions overnight at 4 °C, with gentle shaking. The following primary antibodies were used: rabbit-anti-caspase-3 (1 : 500; (Asp175); Cell Signaling Technology, Danvers, MA, USA); rabbit-anti-caspase-6 (1 : 500; p10 (H-60); Santa Cruz Biotechnology, Dallas, TX, USA) and rabbit-anti-caspase-8 (1 : 500; p18 (H-134); Santa Cruz Biotechnology). Primary antibodies were dissolved in 1% milk in TBS-T. Following primary antibody incubation, blots were washed three times for 15 min each time in TBS-T and incubated in a 1 : 2000 dilution of secondary antibody (horseradish peroxidase conjugated, cross-reacted against rat serum antigens; Jackson ImmunoResearch, West Grove, PA, USA) dissolved in 5% milk in TBS-T. Afterwards, the membranes were rinsed three times for 15 min each time in TBS-T and visualized. Chemiluminescent immunoreactive complexes were visualized using a Bio-Rad Fluor-S Max imager. Loading was verified by re-probing blots with antisera directed against GAPDH (1 : 1000; rabbit polyclonal; Cell Signaling Technology). For quantification, the optical density of each band was normalized against the density of the corresponding GAPDH band for each lane. The normalized densitometry values for each experimental group were reported as mean±S.E.M.

### Colorimetric detection of caspase activity

We examined the effect of caspase-6 and -8 inhibitors on the activity of CASP3 and CASP6 in the ischemic brain or retina. Animals were randomized and divided into three groups (four per group); treatment was the same as described in the caspase western blots section. This assay utilizes a synthetic tetrapeptide, Asp-Glu-Val-Asp (DEVD) to detect caspase-3 cleavage and Val-Glu-Ile-Asp (VEID) to detect caspase-6 cleavage. Colorimetric detection of CASP3 and CASP6 activity was performed using ab39401 CASP3 and ab39709 CASP6 colorimetric assays kit (Abcam, Toronto, ON, Canada). DEVD or VEID-dependent protease activity was assayed by spectrophotometric detection of the free *p*NA cleaved from the substrates. The *p*NA light emission was quantified by a multi-well plate absorbance reader (Sunrise—Tecan, Morrisville NC, USA) at a wavelength of 405 nm. Comparison of the absorbance of *p*NA from caspase-6 or -8 inhibitor-treated samples with control allowed determination of the fold increase in CASP3 and CASP6 activity. The absorbance values at 405 nm for each experimental group were reported as mean±S.E.M.

### Neurofilament staining

The effect of caspase-6 and -8 inhibitors on levels of neurofilament (NF-200 or NF-H; 200 kDa) in the peri-infarct area of the injured hemisphere was examined in a separate group of experimental animals (*n*=6 for each treatment), at 7 days after MCAO.

Following intracardial perfusion, brains were collected and sectioned using a cryostat microtome. Immunohistochemistry for NF-200 was performed using a rabbit-anti-NF-200 primary antibody (N4142, Sigma-Aldrich, St. Louis, MO, USA). Nine sections (14 *μ*m thickness) from the peri-infarct area of the injured hemisphere were visualized using fluorescence microscopy. Sections were taken 1 mm apart starting at 3.70 mm anterior to the bregma. The mean number of NF-200 labeled cell bodies per mm^2^ was calculated and reported as a ratio relative to control ±S.E.M.

### Ki-67 staining after MCAO

To evaluate cell proliferation, Ki-67, a marker of proliferating cells,^27^ was immunolabeled in transverse frozen sections of rat brain, at 7 days after MCAO (Anti-Ki-67, 1 : 300, Abcam). Control, Z-VEID-FMK, or Z-IETD-FMK treatments were compared (*n*=6 for each group). The data were reported as a ratio of mean percentage of Ki-67 positive nuclei/DAPI (mean%±S.E.M.%).

### Statistical analysis

The data for NF-200 immunopositive cell numbers, Ki-67 positive percentages, RGC densities (grouped by retinal eccentricity- inner, middle, outer), RBPMS immunopositive cells densities, and the absorbance values of colorimetric assays were presented as mean±S.E.M. Statistical significance between groups was calculated by performing an analysis of variance (one-way ANOVA) followed by the Tukey's *post hoc* tests. Statistical significance with respect to brain infarct volume, water content or edema percentage between experimental and control groups was evaluated using the Dunnett Multiple Comparisons Test following an ANOVA. Neurological deficits and seizure activity were reported as median and interquartile ranges (25th and 75th percentiles) and were analyzed using the Kruskal–Wallis and Wilcoxon signed rank test. For western blots, normalized densitometry values for each experimental group were reported as mean±S.E.M., and statistically significant differences between experimental and control groups were calculated using a welch-corrected, unpaired *t*-test. Differences were considered significant when *P*<0.05.

## Figures and Tables

**Table 1:** Neurological deficit scores according to the modified Bederson's scoring system

**Group**	**Base**	**2 h**	**8 h**	**24 h**	**48 h**
Ctrl	0 (0–0)	3 (3–4)	3 (3–4)	3 (2–4)	3 (2–3)
Z-VEID	0 (0–0)	3 (2–4)	3 (2–3)	2 (1–3)	2 (1–2)^a^
Z-IETD	0 (0–0)	3 (3–4)	3 (2–3)	2 (2–3)	2 (1–2)^a^

a Significantly different from control group (*P*<0.05). Data are presented as median interquartile range (25–75%)

## Abbreviations

*CCA*: common carotid artery
*CNS*: central nervous system
*DMSO*: dimethyl sulfoxide
*ECA*: external carotid artery
*GAPDH*: glyceraldehyde 3-phosphate dehydrogenase
*ICA*: internal carotid artery
*MCA*: middle cerebral artery
*MCAO*: middle cerebral artery occlusion
*NF*: neurofilament
*RBPMS*: RNA-binding protein with multiple splicing
*RGC*: retinal ganglion cell
*ROCK*: rho-associated protein kinase
*siRNA*: small interfering RNA
*TTC*: triphenyltetrazolium chloride
*Z-IETD-FMK*: Z-Ile-Glu(OMe)-Thr-Asp(OMe)- fluoromethylketone
*Z-VEID-FMK*: Z-Val-Glu-Ile-Asp-fluoromethylketone
